# Preclinical effects of CRLX101, an investigational camptothecin-containing nanoparticle drug conjugate, on treating glioblastoma multiforme via apoptosis and antiangiogenesis

**DOI:** 10.18632/oncotarget.9878

**Published:** 2016-06-07

**Authors:** Chien-Ju Lin, Yi-Ling Lin, Frank Luh, Yun Yen, Ruei-Ming Chen

**Affiliations:** ^1^ Graduate Institute of Medical Sciences, College of Medicine, Taipei Medical University, Taipei, Taiwan; ^2^ Brain Disease Research Center, Taipei Medical University-Wan Fang Hospital, Taipei, Taiwan; ^3^ Sino-American Cancer Foundation, Temple City, California, USA; ^4^ Comprehensive Cancer Center, Taipei Medical University, Taipei, Taiwan; ^5^ Program for Cancer Biology and Drug Discovery, College of Medical Science and Technology, Taipei Medical University, Taipei, Taiwan; ^6^ Anesthetics and Toxicology Research Center and Department of Anesthesiology, Taipei Medical University Hospital, Taipei, Taiwan

**Keywords:** nanoparticle, malignant glioma, apoptosis, angiogenesis

## Abstract

Malignant gliomas are difficult to treat in clinical practice. This study was aimed to investigate the preclinical efficacy of CRLX101, an investigational nanoparticle-drug conjugate developed by conjugating camptothecin (CPT) with cyclodextrin-polyethylene glycol, against gliomas. CPT fluorescence was detected across tight-junction barriers and in mouse plasma and brain. Following CRLX101 treatment, CPT was distributed in the cytoplasm of human U87 MG glioma cells. U87 MG cell viability was decreased by CRLX101 and CPT. Moreover, CRLX101 induced less cytotoxicity to human astrocytes compared to CPT. Exposure of U87 MG cells to CRLX101 induced G_2_/M cell cycle arrest and apoptosis. Administration of CRLX101 induced apoptosis in mice brain tumor tissues and prolonged the survival rate of mice. In addition, CRLX101 inhibited hypoxia and angiogenesis by suppressing the expression of carbonic anhydrase IX, vascular endothelial growth factor, and CD31 in tumor sections. Taken together, this preclinical study showed that CRLX101 possesses antitumor abilities by inducing cell cycle arrest and apoptosis in glioma cells and inhibiting tumor angiogenesis, thereby prolonging the lifespan of mice bearing intracranial gliomas. These data support further research of CRLX101 in patients with brain tumors.

## INTRODUCTION

Grade IV glioblastoma (GBM) is the most common primary brain tumor [1]. GBM is highly mobile and invasive with a poor prognosis and high mortality [2]. Even with combined surgical and medical therapies, the median survival of GBM patients is only 1 year [3]. Despite advances in aggressive treatment, malignant gliomas remain a fatal disease. The poor outcomes are because of uncontrolled tumor proliferation, infiltrative growth, angiogenesis, and resistance to apoptosis [4]. In addition, the blood-brain barrier (BBB) is a major limitation for therapy of GBM. The BBB is constructed by cerebral endothelial cells (CECs), astrocytes, and pericytes [5]. CECs form the tight junctions to limit penetration of most drugs into brain tumors [6, 7]. Thus, developing effective chemotherapeutic agents for brain cancer therapy remains a challenge.

Camptothecin (CPT) is an anticancer drug which inhibits topoisomerase I (Topo-I) [8]. However, CPT has therapeutic limitations because of its poor water solubility and an inactive form of CPT through an E-ring opening reaction [9]. Although water-soluble analogs of CPT, such as topotecan and irinotecan, were approved, these drugs still exhibit sub-optimal pharmacokinetics and have dose-limiting toxicities [10]. Recently, CPT-loaded mesoporous silica nanoparticles and amphophilic cyclodextrin nanoparticles revealed their significant tumor-suppression effect in pancreatic cancer xenografts and rat glioma model, respectively [11, 12]. These studies demonstrate that nanoparticle-based drug delivery system is beneficial to enhancing drug efficacy.

CRLX101 (Cerulean Pharma, Cambridge, MA) is a nanoparticle-drug conjugate (NDC), containing approximately 10 wt% CPT conjugated to a linear, cyclodextrin-polyethylene glycol (CD-PEG) copolymer [13, 14]. Thecyclodextrin-containing polymer is conjugated with CPT by covalent linkage to enhance its water-solubility, and this physical integrity can self-assemble into a nanoparticle of approximately 20~30 nm diameter [13]. A rat pharmacokinetic study showed that the plasma concentrations and the area under the curve of polymer-conjugated CPT are higher than those of CPT alone [15]. The intratumoral concentration of CPT harvested from CRLX101-treated mice was also higher than that with CPT administration in human LS174T colorectal cancer xenografts [13, 15]. Therefore, the enhanced pharmacokinetics and distribution profiles of CRLX101 augment the efficacy of CPT [16]. CRLX101 exhibited anticancer activity in various cancer xenografts, such as colorectal, breast, and pancreatic cancers [9, 16] and is currently in phase 2 clinical trials for metastatic renal cell carcinoma (NCT02187302) and recurrent ovarian cancer (NCT01652079) in combination with bevacizumab, and in phase 1b/2a in combination with chemoradiation in neoadjuvant rectal cancer (NCT02010567). However, the effects of CRLX101 on brain cancer therapy remain unclear.

Hypoxic regions are frequently found in GBM and linked to cell proliferation, cell death, invasion, metastasis, and poor prognoses [17]. Carbonic anhydrase IX (CA IX) is a transmembrane glycoprotein to maintain the acid-base balance and intercellular communication [18]. CA IX is considered a marker of hypoxia and is a hypoxia-inducible factor (HIF)-1α target gene [19, 20]. Angiogenesis is a key process of forming new blood vessels during tumor growth, invasion, and metastasis during hypoxic conditions [21]. Vascular endothelial growth factor (VEGF), a major paracrine mediator in the pathogenesis of glioblastomas and tumor angiogenesis [22, 23], is also induced by HIF-1α [24]. VEGF-targeting agents can inhibit tumor angiogenesis and growth in various cancers [25]. The topoisomerase I inhibitors, topotecan and CPT analogs, were shown to be HIF-1α inhibitors that decrease angiogenesis [26]. CRLX101 has also been shown to inhibit HIF-1α preclinically [27]. A pilot trial of oral topotecan in patients with advanced solid tumors showed that it decreased HIF-1α expression [28]. In view of the fact that suppression of angiogenesis and hypoxia are critical steps in developing curative therapy for GBM, we investigated the preclinical efficacy of CRLX101 against GBM. We revealed for the first time the anticancer efficacy of CRLX101 in glioma cell lines and in an orthotopic intracranial glioma model.

## RESULTS

### The activity of CPT passing through a tight-junction barrier and the distribution of CRLX101 in cells

**Figure 1:**
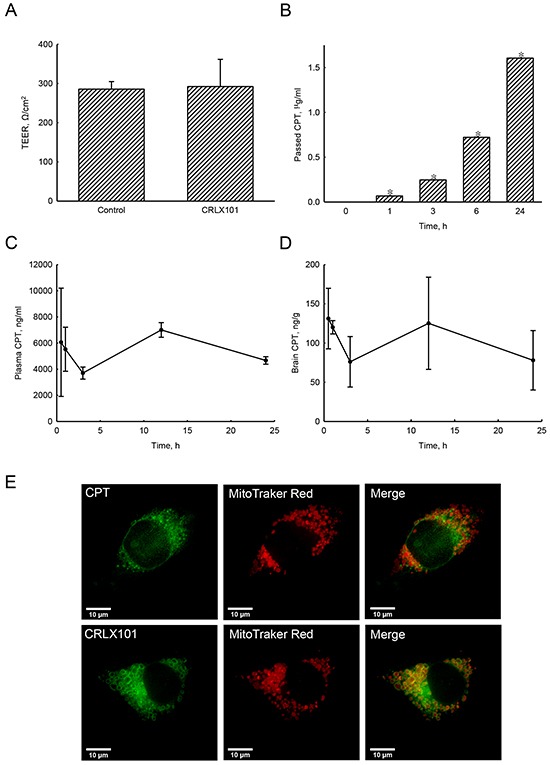
Effects of CPT fluorescence across tight-junction barrier and cell membranes following CRLX101 treatment Tight-junction barrier was constructed by CECs. CRLX101 (30 μg/ml) was added to the upper chamber for the indicated time intervals. The permeability of the CEC monolayer was determined by a TEER assay after 24 hours **A.** The bottom medium was collected to measure the fluorescence of CPT. Concentrations were determined by comparison to a CRLX101 standard curve **B.** Each value represents the mean ± SE for *n*=3. *, *p*<0.05, compared with respective control. Mice were intravenously injected with 10 mg/kg CRLX101 for various time intervals. CPT fluorescence of was evaluated, and concentrations in the plasma **C.** and brain **D.** were quantified with a CRLX101 standard curve. Each value represents the mean ± SE for *n*=3. U87 MG cells were treated with CPT or CRLX101 for 6 hours and stained with Mitotracker Red. The fluorescence was detected using a deconvolution microscope **E.** Data shown are representative fluorescent micrographs of three independent experiments. Scale, 10 μm.

To determine whether CRLX101 treatment results in measurable CPT concentrations across a tight-junction barrier, an *in vitro* BBB model was established. After constructing a tight-junction barrier, data from the TEER assay showed the integrity of the tight-junction barrier (Figure 1A). Addition of CRLX101 for 24 hours did not affect the permeability or integrity of this barrier (Figure 1A). After CRLX101 was added to the upper layer of a transwell for 0~24 hours, the concentration of CPT passing through the tight junction barrier increased in a time-dependent manner (Figure 1B). In addition, after intravenous injection of CRLX101 into ICR mice, CPT fluorescence could be stably detected at 0.5-24 hours post-administration with concentrations ranging from 3702±378 to 7003±796 ng/ml in plasma (Figure 1C) and 76±26 to 131±32 ng/g in brain tissues (Figure 1D). To evaluate whether CRLX101 can enter cells as intact nanoparticles, U87 MG cells were treated with CPT or CRLX101 for 6 hours and then stained with Mitotracker Red to approximately indicate the shape of cells. Although both fluorescent drugs were located inside the cell cytoplasm, stronger fluorescence was found in CRLX101-treated cells compared to CPT-treated cells (Figure 1E). Furthermore, following CRLX101 treatment, CPT was evenly distributed in the entire cell cytoplasm; whereas following CPT treatment, less CPT was distributed in the cytoplasm (data not shown).

### *In vitro* cytotoxicity of CRLX101 against glioma cells and astrocytes

**Figure 2:**
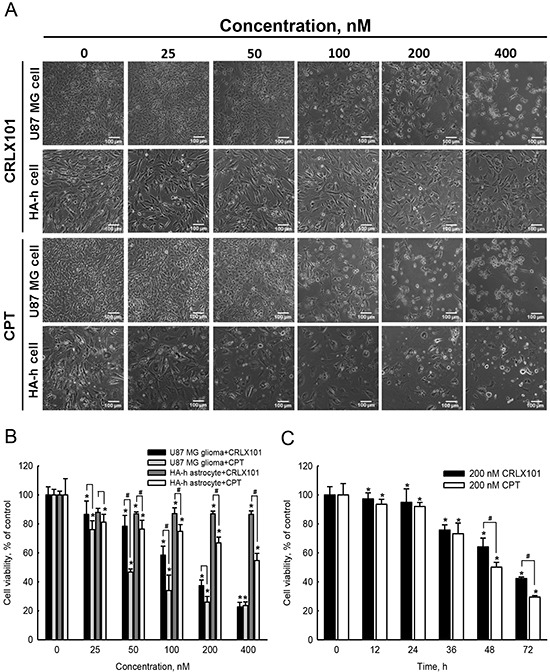
*In vitro* cytotoxicity of CRLX101 against human glioma cells and normal astrocytes Human U87 MG cells and normal HA-h astrocytes were treated with 25~400 nM CRLX101 or CPT for 72 hours. Cell morphologies were observed and photographed using a reverse-phase microscope **A.** Photographs were taken at 100x magnification, and representative sections are shown. Scale, 100 μm. Viability of U87 MG and HA-h cells were assessed using an MTT assay **B.** Human U87 MG cells were treated with 200 nM CRLX101 or CPT for 0~72 hours, and cell viability was assessed using an MTT assay **C.** Each value represents the mean ± SE for *n*=6. *, *p*<0.05, compared with respective control. ^#^, *p*<0.05, compared with CPT group.

Treatment of U87 MG cells with 25~400 nM CRLX101 or CPT for 72 hours decreased cell numbers and caused cell shrinkage (Figure 2A, top and third rows). CRLX101 only slightly inhibited the growth of normal HA-h astrocytes (Figure 2A, second row). However, administration of CPT caused shrinkage and cell numbers in HA-h astrocytes (Figure 2A, bottom row). Both CRLX101 and CPT decreased the viability of U87 MG cells in dose-dependent manner (Figure 2B). HA-h cell viability was sustained at around 87% after treatment with 25~400 nM CRLX101. However, CPT suppressed viability of human HA-h cells more significantly compared to CRLX101 (*p*<0.05) (Figure 2B). The 50% inhibitory concentration (IC_50_) of CRLX101 and CPT on U87 MG cells were 204 and 142 nM, respectively. Thus, a dose of 200 nM was chosen for use in the following experiments. After treatment of U87 MG cells with 200 nM CRLX101 or CPT for 12 to 72 hours, cell viability decreased over time (Figure 2C).

### Mechanisms of cell growth inhibition by CRLX101 in glioma cells

**Figure 3:**
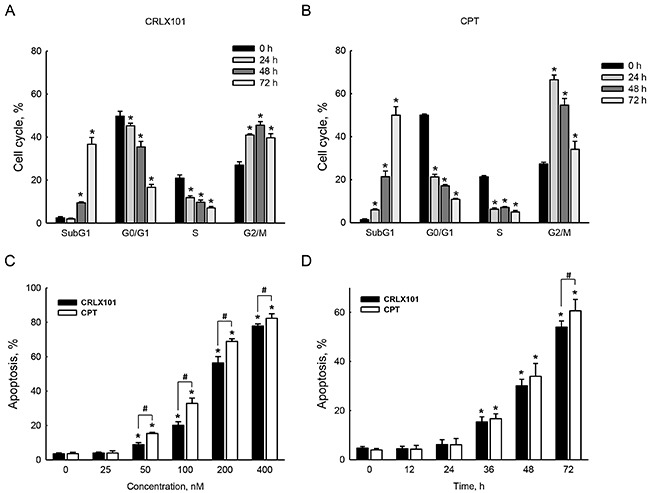
Effects of CRLX101 on inducing cell cycle arrest and cell death U87 MG cells were exposed to 200 nM CRLX101 **A.** or CPT **B.** for 24, 48, and 72 hours. The cell cycle was analyzed using flow cytometry. U87 MG cells were treated with 25~400 nM CRLX101 or CPT for 72 hours **C.** or were treated with 200 nM CRLX101 or CPT for the indicated time periods **D.** Modes of cell death were analyzed and quantified as described in “Materials and Methods”. Each value represents the mean ± SE for *n*=3. *, *p*<0.05, compared with respective control. ^#^, *p*<0.05, compared with CPT group.

To investigate whether the cell cycle was affected by CRLX101, DNA contents of cells were detected using flow cytometry with PI staining. Treatment with 200 nM CRLX101 induced cell cycle arrest in the G_2_/M phase from 24 to 72 hours and increased percentage of cells in the subG_1_ phase at 48 and 72 hours (Figure 3A). In parallel, CPT increased percentages of cells in the G_2_/M and subG_1_ phases from 24 to 72 hours (Figure 3B). The percentage of total apoptosis significantly increased after U87 MG cells were treated with 50~400 nM CRLX101 and CPT for 72 hours (Figure 3C). However, neither CRLX101 nor CPT treatment induced necrosis (data not shown). Exposure of U87 MG cells to 200 nM CRLX101 and CPT induced apoptosis from 36 to 72 hours (Figure 3D) without necrosis (data not shown).

### *In vivo* efficacy of CRLX101 in intracranial gliomas

**Figure 4:**
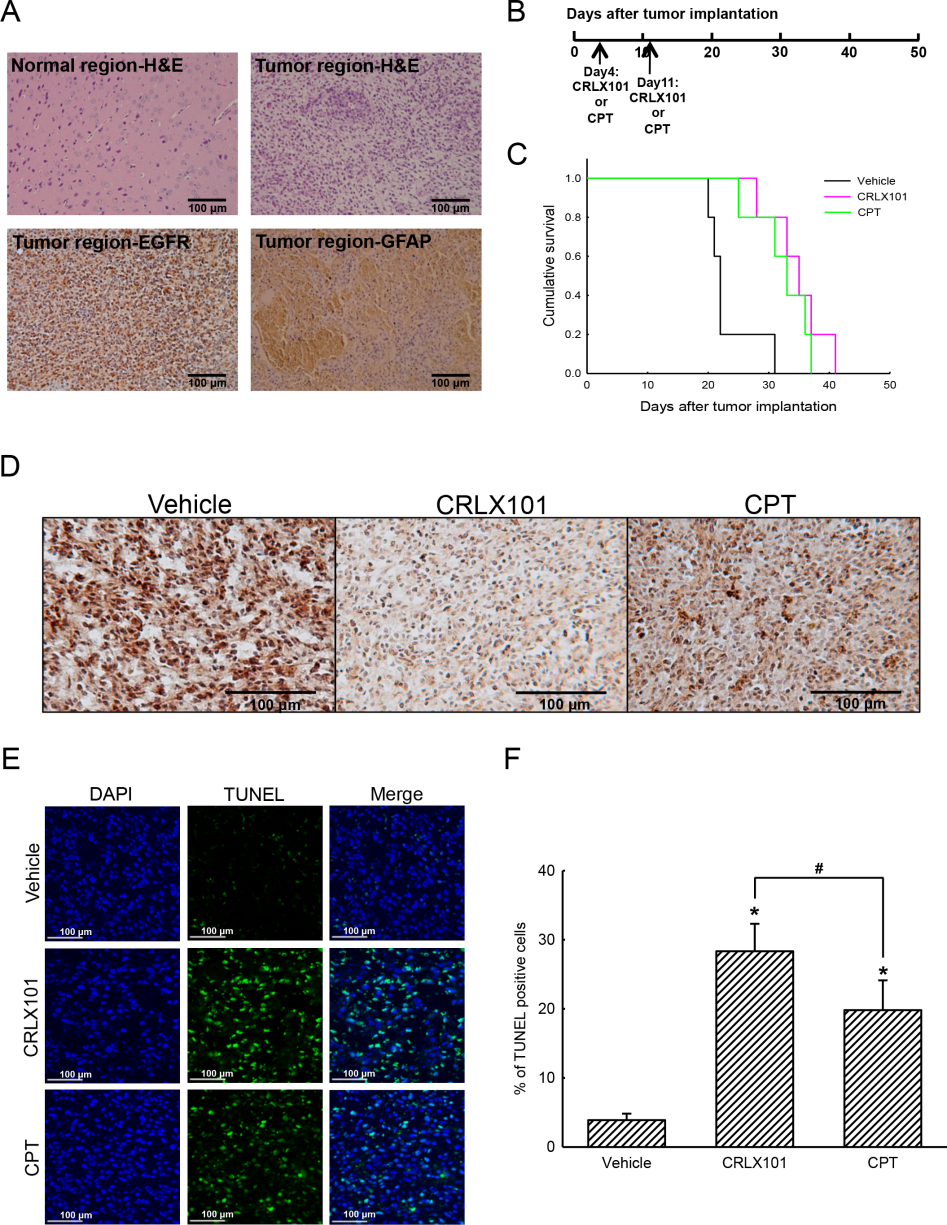
*In vivo* anticancer efficacy of CRLX101 against brain tumors Brain tissues were removed and sectioned for H&E staining and IHC analyses of EGFR and GFAP **A.** Time line of CRLX101 and camptothecin (CPT) administration in nude mice bearing intracranial U87 MG gliomas **B.** A Kaplan-Meier survival curve was analyzed by a log-rank test **C.** Expression levels of topoisomerase I (Topo-I) in tumor sections were analyzed by IHC, and representative images are shown **D.** A TUNEL assay was performed on sections from each group of mice. Representative images are shown **E.** TUNEL-positive cells were quantified and are expressed as percent stained per field **F.** Each value represents the mean ± SE. *, *p*<0.05, compared with respective control. ^#^, *p*<0.05, compared with CPT group. Scale, 100 μm.

The antitumor efficacy of CRLX101 was also evaluated in an animal brain tumor model. The histological analysis with H&E staining showed no tumor in the left control hemisphere (Figure 4A, upper-left panel), whereas the tumor had grown in the right hemisphere of the brain (Figure 4A, upper-right panel). Using IHC to identify GBM, we demonstrated that the EGFR (Figure 4A, lower-left panel), and the astrocytic marker, GFAP (Figure 4A, lower-right panel) were present in brain tumor sections. In order to reveal the efficacy of CRLX101, tumor-bearing mice were injected with 10 mg/kg CRLX101 or CPT once/week for 2 weeks 4 days after tumor implantation. The time line for the administration of CRLX101 or CPT is depicted in Figure 4B. The survival rate was analyzed after the mice expired naturally or were euthanized. Median survival times of mice without treatment and following treatment with CRLX101 or CPT were 22, 35, and 32 days, respectively. CRLX101 prolonged the survival rate of mice as assessed by a Kaplan-Meier survival curve and analyzed using a log-rank test (Figure 4C). In addition, administration of CRLX101 or CPT decreased Topo-I expression in tumor sections (Figure 4D). Compared to the vehicle and the CPT treatment groups, administration of CRLX101 significantly increased number of TUNEL-positive glioma cells in brain tumor tissues (Figure 4E and 4F).

### *In vitro* and *in vivo* antiangiogenic effects of CRLX101

**Figure 5:**
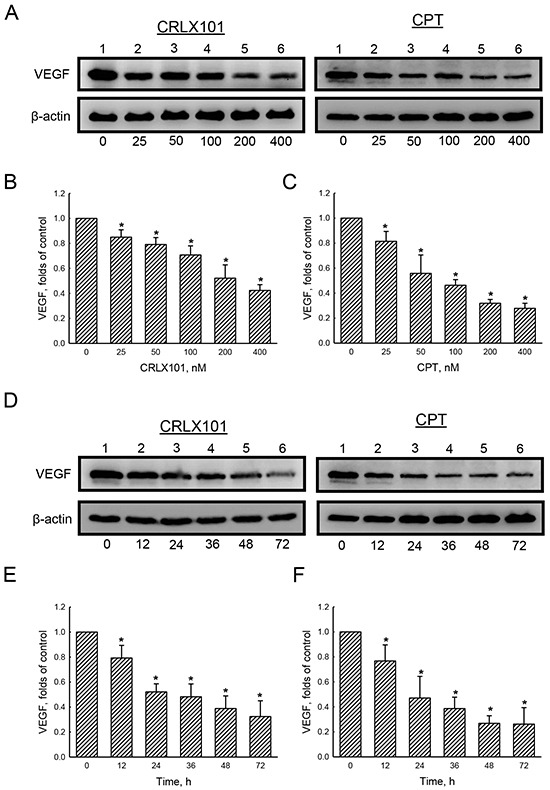
*In vitro* effect of CRLX101 on levels of VEGF U87 MG cells were treated with 25~400 nM of CRLX101 or camptothecin (CPT) for 72 hours **A-C.** or with 200 nM CRLX101 or CPT for 12~72 hours **D-F.** Cell lysates (50 μg per lane) were analyzed using immunoblotting. Representative data are shown in panel A and D. These protein bands were individually quantified and analyzed in panel B, C, E, and F. Each value represents the mean ± SE for *n*=3. *, *p*<0.05, compared with respective control.

**Figure 6:**
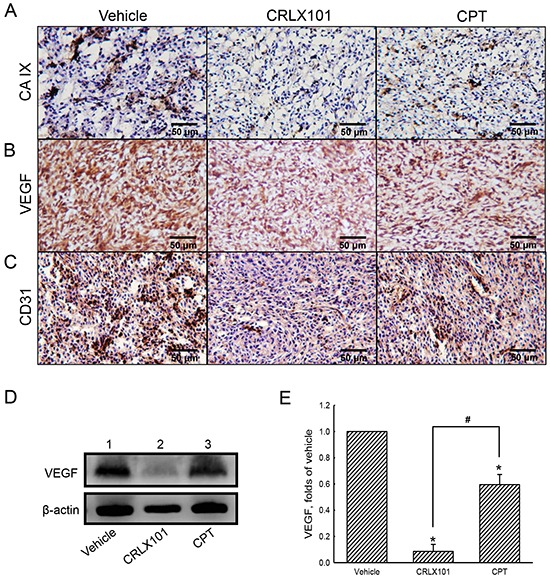
Antiangiogenic effect of CRLX101 in mice intracranial gliomas After administration of CTLX101 or camptothecin (CPT), tumor sections from mice with intracranial gliomas were analyzed using IHC of CA IX **A.** VEGF **B.** and CD31 **C.** Representative images of tumor sections are shown. Scale, 50 μm. **D.** Cell lysates (50 μg per lane) extracted from tumors was analyzed using immunoblotting. These protein bands were quantified and analyzed in panel **E.** Each value represents the mean ± SE for *n*=3. *, *p*<0.05, compared with respective control. ^#^, *p*<0.05, compared with CPT group.

The expression of VEGF was examined using immunoblotting to reveal the effect of CRLX101 on angiogenesis. Protein levels of VEGF decreased after U87 MG cells were treated with 25~400 nM CRLX101 (Figure 5A and 5B) or CPT (Figure 5A and 5C) for 72 hours. Exposure of U87 MG cells to 200 nM CRLX101 or CPT suppressed protein levels of VEGF in time-dependent manners (Figure 5D-5F). In the *in vivo* intracranial glioma model, the expression of CA IX, a marker of hypoxia, was highest in the vehicle group; administration of CRLX101 decreased CA IX expression more than CPT administration (Figure 6A). Subsequently, VEGF expression in tumor region was suppressed by CRLX101 and CPT (Figure 6B). Both CRLX101 and CPT treatments resulted in decreased CD31 expression compared to the vehicle group (Figure 6C). In addition, CRLX101 markedly reduced the protein level of VEGF compared to the vehicle and CPT groups (Figure 6D and 6E).

## DISCUSSION

A preclinical study showed better efficacy with CRLX101 than CPT-11 in inhibiting DNA Topo-I catalytic activities and prolonging the survival of lymphoma xenograft-bearing mice [29]. Emerging clinical data suggest that CRLX101 is generally well tolerated in cancer patients across several indications [16], with early clinical studies showing encouraging tolerability, pharmacokinetic, and efficacy results [30]. Our study is the first report of the preclinical efficacy of CRLX101 in gliomas using both cell and animal models. In addition, we have also shown the efficacy of CRLX101 on killing human U373MG glioma cells (data not shown). Thus, this study demonstrated that CRLX101 has preclinical anticancer activities via (1) induction of cell cycle arrest and apoptosis, (2) inhibition of angiogenesis in U87 MG glioma cells and in mouse brain tumor tissues, and (3) increase in survival rate for mice with intracranial gliomas.

The BBB tight junctions limit the number of therapeutic drugs entering the brain [27]. A study showed that, after administration of CRLX101, concentrations of released CPT in tumor tissues harvested from LS174T colorectal cancer xenograft mice were higher than in the other tissues, such as the liver, spleen, lungs, and heart [8]. In our *in vitro* BBB model, CPT was detected in the bottom of the transwells. Moreover, our pharmacokinetic analyses showed similar and extended time course patterns for levels of CPT in the plasma and brains of ICR mice intravenously injected with CRLX101. Schluep et al. reported a longer mean elimination half-life for polymer-conjugated CPT than for CPT injection in multiple cancer models [9]. However, this study has no evidence showing penetration of CRLX101 into the BBB. A previous study demonstrated that intact CRLX101 nanoparticles can enter PC-3 cells and release CPT intracellularly [28]. In addition, our data confirmed uniform drug distribution in the cytoplasm of U87 MG cells treated with CRLX101. Our results showed that the CPT component of CRLX101 can cross the tight-junction barrier, retain in brain tissues, and enter glioma cells. Therefore, CRLX101 should be explored further as a potential agent for brain cancer therapy.

In this study, CRLX101 suppressed the cell viability of U87 MG cells in dose- and time-dependent manners. The IC_50_ of CRLX101, when treating U87 MG cells, was 204.1 nM, which is reasonable compared to previous studies with IC_50_ concentrations for lymphoma cells and gastric cancer cells [29, 31]. Our results are consistent with previous studies, reporting lower *in vitro* potency of CRLX101 due to slow CPT release from CRLX101 [10, 29]. Importantly, CRLX101 had higher cytotoxicity in U87 MG cells than in normal HA-h astrocytes. In contrast, CPT damaged more HA-h astrocytes than CRLX101. CPT, a Topo-I inhibitor can induce G_2_/M cell cycle arrest in human colorectal carcinoma cells, human glioblastoma cell lines, neuroblastoma cells, and breast cancer cells [32–35]. Herein, we showed that CRLX101 significantly induced G_2_/M cell cycle arrest and apoptosis in U87 MG cells. Administration of CRLX101 or CPT prolonged the survival rate of mice with intracranial gliomas. CRLX101 suppressed the expression of Topo-I and increased apoptosis in tumor tissues compared to the vehicle or CPT groups. Thus, CRLX101 possesses antitumor effects against gliomas by inducing apoptotic cell death. Our results suggest that CRLX101 provides a key advantage for the development of clinical brain tumor therapy.

Angiogenesis is activated by the HIF-1α/VEGF pathway in hypoxic tumor microenvironments [36]. Antiangiogenic therapy is often evaluated in combination with chemotherapy for treating various cancers [25]. However, some patients do not respond to antiangiogenic therapy or develop resistance due to intratumor hypoxia caused by antiangiogenic agents. Therefore, combining antiangiogenic and hypoxia-targeted therapies was investigated to improve clinical outcomes [37, 38]. A previous report suggested that CRLX101 treatment inhibited the proliferation and angiogenesis of tumor from breast cancer patients [39]. Our results demonstrate that CRLX101 down-regulated expression of VEGF *in vitro* and *in vivo*, and decreased CD31 in tumor sections. Meanwhile, the expression of CA IX, a biomarker of hypoxia, also decreased in tumor tissues. Therefore, our study suggests a promising effect of CRLX101 in inhibiting hypoxia and angiogenesis. Moreover, our *in vivo* results indicate that CRLX101 was more effective than CPT in inducing apoptosis and suppressing angiogenesis due to CRLX101's improved drug delivery profile and enhanced permeability and retention effect [40]. Clinically, the combination of CRLX101 plus the VEGF antibody bevacizumab is currently being evaluated in metastatic renal cell carcinoma and recurrent ovarian cancer. Further improvement in efficacy may be possible by combining CRLX101 with antiangiogenic therapy.

In conclusion, this study showed that the CPT component from CRLX101 could pass CEC-constructed tight-junction barriers and BBB in mice, and locate in U87 MG cells. CRLX101 decreased the viability of U87 MG cells through inducing cell cycle arrest and apoptosis, but not necrosis. In our *in vivo* brain tumor model, CRLX101 prolonged the survival rate of nude mice with intracranial gliomas by inducing apoptosis. In addition, CRLX101 had an antiangiogenic effect *in vitro* and *in vivo* through suppressing the HIF-1α/VEGF pathway. Taken together, our results suggest that CRLX101 may be a promising novel agent for brain tumor therapy; further research is warranted. However, there are certain study limitations in this study, including the penetration of CRLX101 into the BBB and its effects on tumor growth. We will use mass spectrophotometers and the Xenogen IVIS-200 System to determine these two effects in our upcoming study.

## MATERIALS AND METHODS

### Cell culture and drug treatment

The U87 MG human glioblastoma cell line purchased from the American Type Culture Collection (Manassas, VA) was maintained in Minimum Essential Media (MEM; Gibco-BRL Life Technologies, Grand Island, NY) supplemented with 10% fetal bovine serum (FBS), 2 mM L-glutamine, 100 IU/mL penicillin, 100 mg/mL streptomycin, 1 mM sodium pyruvate, and 1 mM nonessential amino acids at 37°C in a humidified atmosphere of 5% CO_2_. Human astrocytes (HA-h) from ScienCell Research Laboratories (San Diego, CA) were cultured in astrocyte medium (ScienCell Research Laboratories). Cells were grown to confluence before drug treatment. CRLX101 was provided by the Cerulean Pharma (Waltham, MA). The physical characteristics of CRLX101 and its detailed assembly and disassembly were described previously [13]. CRLX101 was dissolved in sterile double-distilled H_2_O. CPT purchased from Sigma (St. Louis, MO) was dissolved in dimethyl sulfoxide (DMSO).

### Determination of transport through CEC tight junctions

Isolation of mouse CECs and construction of tight junctions were prepared according to a previously described method [41]. Briefly, 10^6^ CECs were seeded in Transwell cell culture chamber inserts (BD Biosciences, Franklin Lakes, NJ) for 4 days to form tight-junction barriers. CRLX101 (30 μg/ml) was added to the top chambers for 1, 3, 6, and 24 hours. After drug treatment, medium from the bottom chamber was collected. Fluorescence readings (excitation at 370 nm and emission at 440 nm) of the measured CPT were analyzed using a fluorescence spectrometer (PerkinElmer, Waltham, MA).

### Measurement of the transendothelial electrical resistance (TEER)

Permeability of the CEC monolayer was assayed by determining the TEER using an EVOM resistance meter (World Precision Instruments, Sarasota, FL) following a previously described method [42]. The corrected TEER value was calculated by subtracting the resistance of a blank filter from the monolayer and is expressed as Ω/cm^2^.

### Animal treatment for drug distribution

All procedures were performed according to the *Guide for the Care and Use of the Laboratory Animals* published by the US National Institutes of Health (NIH Publication no. 85-23, revised 1996) and approved by the Institutional Animal Care and Use Committee of Taipei Medical University (Taipei, Taiwan). Male ICR mice were purchased from the Animal Center of the College of Medicine, National Taiwan University (Taipei, Taiwan). Animals were intravenously injected with a single dose of 10 mg/kg CRLX101 following a previous method [29]. Previous studies have shown the safeties of the empty CD-PEG nanoparticles at a dosage up to 240 mg/kg in mice [43, 44]. Mice were sacrificed and blood was collected at 0.5, 1, 3, 12, and 24 hours post-dose and plasma was separated from the blood. Acetonitrile was added to the plasma, and the mixture was centrifuged at 10000 rpm for 10 min to obtain the supernatant. Animals were sacrificed after perfusion. Brain tissues were homogenized and extracted with acetonitrile. To determine the CPT concentration, the fluorescence of CPT in the supernatant from brain tissues and plasma and standard CRLX101 were measured at 370 nm for excitation and 440 nm for emission using a fluorescence spectrometer (PerkinElmer). Concentrations were calculated with a standard curve.

### Microscopic analysis of drug distributions

U87 MG cells were seeded on coverslip for 24 hours and treated with CRLX101 or CPT for 6 hours. Cells were stained with MitoTracker^®^ Red CMXRos dye (Mitotracker Red, Life Technologies, Gaithersburg, MD) to label mitochondria in the cytosol. Fluorescence images of CPT and Mitotracker Red were captured with a wide-field DeltaVision deconvolution microscope (Applied Precision, GE Healthcare Life Science, Pittsburgh PA), equipped with a 60x/1.42 N.A. oil-immersion objective lens. Both the microscope and camera were controlled by SoftWorX application suite software. Stacks of optical section images, with an image size of 1024 × 1024 pixels, were collected for all fluorochromes. All images were deconvolved using SoftWorX software (Applied Precision), and analyzed with VoloCITY software (PerkinElmer).

### Cell viability assay

Cell viability was assayed using 3-(4,5- dimethylthiazol-2-yl)-2,5-diphenyltetrazolium bromide (MTT). U87 MG cells or HA-h astrocytes were seeded on a 96-well plate at 10^4^ cells/well for 24 hours, followed by drug treatment for another 72 hours. Before the end of treatment, 0.5 mg/ml MTT was added to each well for 4 hours. The supernatants were carefully aspirated, and formazan crystals were dissolved using DMSO. The absorbance was measured at 550 nm with a microplate reader (Biochrom, Holliston, MA).

### Detection of apoptosis

Apoptosis was analyzed according to a previous study [43]. After treatment, whole cells were collected in HEPES buffer containing 10 mM HEPES (pH 7.4), 140 mM NaCl, and 2.5 mM CaCl_2_. Cells were subsequently stained with annexin V (2.5 μg/ml) and PI (2 ng/ml) for 20 minutes, followed by analysis on a flow cytometer (Beckman Coulter, Brea, CA). The cytogram of the four quadrants in the figure was used to distinguish normal (annexin V^−^/PI^−^), early apoptotic (annexin V^+^/PI^−^), late apoptotic (annexin V^+^/PI^+^), and necrotic cells (annexin V^−^/PI^+^). The sum of early apoptosis and late apoptosis is presented as total apoptosis.

### Cell cycle analysis

After treatment of U87 MG cells with CRLX101 or CPT for 24, 48, and 72 hours, whole cells were collected and centrifuged at 2000 rpm for 5 min. Pellets were resuspended in phosphate-buffered saline (PBS) and fixed in ice-cold 70% ethanol at 4°C. Cells were then washed with PBS and incubated in PBS containing 0.5 mg/ml RNase A and 40 mg/ml PI at 37°C for 30 min in the dark. The cell cycle was analyzed on a flow cytometer (Beckman Coulter, Brea, CA). Results were further analyzed using WinMDI 2.9 software (http://facs.scripps.edu/software.html).

### Animal orthotopic brain tumor model and drug treatment

Six-week-old female nude mice (BALB/c *nu*/*nu*, National Laboratory Animal Center) were housed in a sterile environment (in a specific pathogen-free room) with a light/dark cycle of 12/12 h and were allowed free access to food and water for 1 week. Animals were anesthetized by inhalation of isoflurane and then were stereotactically inoculated with 2 × 10^5^ U87 MG cells (in 3 μl PBS) into the right frontal lobe (2 mm lateral and 1 mm anterior to the bregma, at 3 mm in depth from the skull base) using a Hamilton syringe (Hamilton, Reno, NV) and a syringe pump (SINGA Technology, Taipei, Taiwan). Intracranial glioma-bearing mice were randomly divided into three groups (*n*=5/group) 4 days after tumor implantation and were treated with intravenous injection of 10 mg/kg CRLX101 or intraperitoneal injection of 10 mg/kg CPT qw x2 according to a previous study [29]. The survival analysis considered death as either expired naturally or euthanized prior to death. A Kaplan-Meier survival analysis was performed at the end of the experiment.

### Histology and immunohistochemistry (IHC)

Mice were sacrificed 20 days after implantation of glioma cells. Brains were removed and fixed in 4% paraformaldehyde in PBS, embedded in paraffin, and sectioned. Sections for the histological analysis were deparaffinized with xylene, rehydrated with a graded alcohol series, followed by antigen target retrieval for 20 minutes, and then stained with hematoxylin and eosin (H&E). For IHC, endogenous peroxidase activity was quenched in a 3% H_2_O_2_ solution. Slides were incubated in blocking solution (Vector Laboratories, Burlingame, CA) for 1 hour. Primary antibodies for the epidermal growth factor receptor (EGFR) (1: 500, GeneTex, Irvine, CA), glial fibrillary acidic protein (GFAP) (1: 250, BD Biosciences, Franklin Lakes, NJ), Topo-I (1:50, Abcam, Cambridge, MA), CA IX (1:50, R&D systems, Minneapolis, MN), VEGF (1:200, Santa Cruz Biotechnology, Santa Cruz, CA), and CD31 (1:75, BD Biosciences, Franklin Lakes, NJ) were incubated at 4°C overnight followed by incubation with biotin-conjugated secondary antibodies, for 1 hour at room temperature. Slides were subsequently detected using a Vectastain ABC kit (Vector Laboratories). DAB (Vector Laboratories) is a substrate for peroxidase. Sections were counterstained with hematoxylin, followed by dehydration in graded alcohols and xylene, with the addition of a cover slip. Photomicrographs were taken at 200x magnification with a Nikon microscope equipped with a digital camera (Nikon, Melville, NY).

### In situ detection of apoptosis

DNA fragmentation in brain tumor tissues was detected using an *In Situ* Cell Death Detection kit, Fluorescein (Roche Applied Science, Indianapolis, IN), based on terminal deoxynucleotidyl transferase-mediated dUTP nick end-labeling (TUNEL) technology following the manufacturer's instructions. Briefly, brain tissue sections were deparaffinized and permeabilized in a permeabilization solution (0.1% Triton X-100 and 0.1% sodium citrate). Sections were then incubated with a TUNEL reaction mixture for 1 hour. After incubation, slides were sealed with mounting medium containing DAPI (Vector Laboratories). Images were captured at 60x magnification using an Olympus FluoView FV10i Confocal laser scanning microscope (Olympus, Tokyo, Japan). Numbers of TUNEL-positive cells and total cells were counted using MetaMorph software in more than 10 random microscopic fields. Results are expressed as a percentage of positive cells to the total number of cells counted.

### Immunoblotting

After treatment, U87 MG cells were washed with PBS and lysed in ice-cold lysis buffer (25 mM HEPES, 1.5% Triton X-100, 0.1% sodium dodecylsulfate (SDS), 0.5 M NaCl, 5 mM EDTA, and 0.1 mM sodium deoxycholate) containing a protease inhibitor cocktail. Protein concentrations were quantified using a bicinchonic acid protein assay kit (Thermo, San Jose, CA). An equal amount of proteins from each group was separated using SDS-polyacrylamide gel electrophoresis (PAGE), followed by transfer to nitrocellulose membranes. Membranes were incubated with a 5% skim milk solution (blocking solution) for 1 hour, and then incubated with anti-VEGF and anti-β-actin antibodies (Santa Cruz Biotechnology, Santa Cruz, CA) at 4°C for 16 hours. Membranes were probed with the appropriate horseradish peroxidase (HRP)-conjugated secondary antibodies for 1 hour at room temperature, and then imaged using a Syngene G:BOX iChemi camera (Syngene, Cambridge, UK) and GeneSnap software (vers. 7.09, Syngene). β-actin was used as an internal control. The density of bands was determined with Gel-Pro Analyzer densitometry software.

### Statistical analysis

Data are presented as the mean ± standard error (SE) from three or six (in the case of the MTT assay) independent experiments. Statistical significance was examined using Student's *t*-test (for two groups) or a one-way analysis of variance (ANOVA; for three or more groups). The survival curve of mice was examined using a log-rank test. A *p* value of <0.05 was considered statistically significant.
